# *Streptomyces* sp SM01 isolated from Indian soil produces a novel antibiotic picolinamycin effective against multi drug resistant bacterial strains

**DOI:** 10.1038/s41598-020-66984-w

**Published:** 2020-06-22

**Authors:** Pulak Kumar Maiti, Sujoy Das, Prithidipa Sahoo, Sukhendu Mandal

**Affiliations:** 1 Laboratory of Molecular Bacteriology, Department of Microbiology, University of Calcutta, 35, Ballygunge Circular Road, Kolkata, 700019 India; 2 The Molecular Recognition Laboratory, Department of Chemistry, Visva-Bharati University, Siksha Bhavana, Santiniketan, Birbhum, West Bengal 731235 India

**Keywords:** Antibiotics, Soil microbiology

## Abstract

A Kashmir Himalayan (India) soil isolate, *Streptomyces* sp. SM01 was subjected to small scale fermentation for the production of novel antimicrobials, picolinamycin (SM1). The production has been optimized which found to be maximum while incubated in AIA medium (pH 7) for 7 days at 30 °C. Seven days grew crude cell-free culture media (50 µL) showed a larger zone of inhibition against *Staphylococcus aureus* compared to streptomycin (5 µg) and ampicillin (5 µg). Extraction, purification, and chemical analysis of the antimicrobial component has been proved to be a new class of antibiotic with 1013 dalton molecular weight. We have named this new antibiotic as picolinamycin for consisting picolinamide moiety in the center of the molecule and produced by a *Streptomyces* sp. In general, the antimicrobial potency of this newly characterized antibiotic found to be higher against Gram-positive organisms than the tested Gram-negative organisms. The MIC of this antimicrobial compound was found to be 0.01 µg/ml for tested Gram-positive organisms and 0.02 to 5.12 µg/ml for Gram-negative organisms. Furthermore, it showed strong growth impairments of several multidrug resistance (MDR) strains, including methicillin-resistant strains of Staphylococci and Enterococci with the MIC value of 0.04 to 5.12 µg/ml and MDR (but methicillin-sensitive) strains of *S. aureus* with the MIC value of 0.084 µg/ml. It also showed anti-mycobacterial potential in higher concentrations (MIC is 10.24 µg/ml). Picolinamycin however did not show toxicity against tested A549 human cell line indicating that the spectrum of its activity limited within bacteria only.

## Introduction

About one out of ten patients are acquiring nosocomial infection as hospitals are the principal source of multidrug-resistant (MDR) pathogens^1^. The most formidable MDR pathogens are methicillin-resistant *Staphylococcus aureus* (MRSA), vancomycin-resistant *Enterococcus* (VRE); vancomycin-resistant *S. aureus* (VRSA) and principal Gram-negative bacteria involved in nosocomial infection like *Klebsiella pneumoniae, Acinetobacter baumanni, Pseudomonas aeruginosa, Enterobacter* sp. which generally are also resistant against major existing antibiotics such as second-generation cephalosporin, fluoroquinolones, penicillin in combination with beta-lactamase inhibitor, third-generation cephalosporin and carbapenem^2–8^. Non-judicial use of antibiotics is thought to be the major factor for antimicrobial resistance^8–10^. More than 100,000 tons of antibiotics have been manufactured annually to combat pathogens^2^. However most of the clinically used antibiotics against nosocomial bacteria remain ineffective due to their acquired resistance^11–14^. From January 2000 to October 2019, only 44 compounds under 7 novel classes have been introduced in clinical pipeline^15^. Among these 27 compounds are synthetic, 14 are derivative of natural product and 3 are from other sources. However the antimicrobials of natural origin are very rare, but these are found to be most suitable against multidrug resistance (MDR) bacteria, as they evolved efficiently to cross the barrier of target bacteria^16,17^. Despite the discovery of new antimicrobials, the rise of antibiotic resistance is very fast^18,19^. Consequently, the urge for novel antibiotics becomes higher especially to fight against super-infections, secondary infections, nosocomial infections, and against the MDR strains. Control of these MDR pathogens can be solved by using another group of microbes as the larger portion of antibacterial agents (at least 3/4) in clinical use are either natural products of the microbial origin or one of their analogs^11,20,21^. Among the microbes, actinobacteria are one of the principal factories for novel antibiotic production because nearly two-third of all known antibiotic found to be derived from them^22,23^. *Streptomyces* alone produces around 80% of the total actinobacteria-derived antibiotics^24–27^. Also among all antibiotic-producing microbes, *Streptomces* accounts for 50–55% of known antibiotics production^28,29^. Streptomycetes are complex filamentous, Gram-positive bacteria with high G + C content and exist in all types of environments. In the soil they contain around 90% of total actinobacteria^30^. In general, actinobacteria produce antibiotics when they need to compete with the neighboring genera. Stress condition might frequently appear through nutrient deprivation when actinobacteria especially *Streptomyces* transform from vegetative mycelial structure to erected sporogenic aerial mycelium^26–32^. Although soil contains diverse species of *Streptomyces*, for isolation of novel antibiotic-producers, the target soil sample needs to be very specific to avoid the re-isolation of the same species and same antibiotics^33^. In our previous study we explained the unique climatic parameters of Kashmir soil for isolation of novel antibiotic-producing actinobacteria and have isolated 135 morphologically different actinobacteria, among which some of them are antibiotic producer^34^. This study delineates about the identification of an isolate, *Streptomyces* sp. SM01. Screening of antimicrobial compounds by primary and secondary methods and further production optimization has been carried out in the present study. Purification of the antimicrobial compound has been done through chromatography followed by structural elucidation through various spectrometry and CHN-analysis. To determine the antimicrobial efficacy, we have estimated the MIC and MBC of the purified compound against several test organisms ranging from non-pathogenic (*Bacillus. stratosphericus* MCC 2251), opportunistic (*Bacillus. cereus* MTCC 1272, *S. aureus* MTCC 96, *Staphylococcus. epidermidis* MTCC 3086, *K. pneumoniae*, *Escherichia. coli* MTCC 1687, *P. aeruginosa*, etc.) and eight MDR strains. We have shown that picolinamycin does not have any cytotoxicity on A549 human cell line indicating its spectrum of activity limited within bacteria.

## Methods

### Isolation of actinomycetes

The soil samples were collected from Rangreth of Kashmir Himalaya, India (latitude: 34°-01′N; longitude: 74°-47′E; altitude: 5328 ft; annual rainfall: 743 mm, average temperature: 13.6 °C). Soil samples were pre-treated with CaCO_3_ for 7 days followed by heat treatment for 2 hr in a hot air oven at 65 °C to enrich and selectively isolate actinobacteria^35,36^. After that 1 g of soil was dissolved in 1 ml of 0.9% NaCl and a serial dilution up to 10^−5^ has been made. From these dilutions, each 0.1 ml of sample was spread on Actinomycetes isolation agar (AIA) medium^37^ supplemented with 50 µg/ml of cycloheximide and nystatin to inhibit the unwanted fungal growth^34^. The inoculated plates were incubated for 3–4 days at 28 °C. Incubated plates showed various actinobacterial colonies. We have isolated 135 strains based on morphometry (substrate and aerial mycelium, soluble pigments, diffusible pigments)^34^ and selectively proceed with isolates having antimicrobial production ability. We have screened them for their unique features which could indicate their novelty as an isolate and their ability to produce non-redundant antimicrobials^34^. SM01 colony has been selected for its unique features, picked by the sterile toothpick, and streaked on fresh (International Streptomyces Project) ISP-2 plate to get pure cultures^37,38^. The culture was preserved for a long time with 20% (v/v) glycerol at −80 °C.

### Identification of SM01

The differential pigmentation (aerial and substrate mycelium and diffusible pigments) of the isolate SM01 was observed after 14 days of incubation on ISP-2 medium^38^. Colony morphology which includes size, shape, margin, texture, form, optical property of the colony, was noticed after 14 days of incubation on ISP-2 medium. Scanning electron microscopy was performed following standard methods to visualize the detailed morphology of SM01^39^. The 16 S rDNA gene was amplified by using universal primer 8 F and 1492 R followed by sequencing and sequence analysis using both the BLASTn program (https://blast.ncbi.nlm.nih.gov) and EZbiocloud server^40^. 16 S rDNA gene sequences of all required type strains were taken from these databanks and phylogenetic analyses were completed by various algorithms like Neighbour Joining (NJ)^41^, Maximum Likelihood (ML)^42^ and Maximum Parsimony (MP)^43^. Thus, both distance-based and character-based phylogenetic trees were constructed to overview the evolutionary status of SM01. The required 16 S rDNA gene sequence was deposited in NCBI and the accession number is MH299817.

### Preliminary screening for antimicrobial property

Antimicrobial activity was assessed by the agar-diffusion method on Mueller-Hinton (MH) agar media^44^. By this method 5–7 days old colony of SM01 was scooped by a sterile loop with about 10 mm in diameter and placed it on plates having the lawn of the test organism. For preliminary assay two Gram-positive (*S. aureus* MTCC 96, *B. cereus* MTCC 1272), two Gram-negative (*E. coli* MTCC 1687, *P. aeruginosa*) bacteria and one yeast (*Saccharomyces cerevisiae*) were used as test organisms. The plates were kept at 4 °C for 2 hrs for a homogenous distribution of antimicrobial compounds and incubated further at 37 °C for overnight^45^. Plates with yeast as test organism have been incubated for 48 hrs at 28 °C. Positive results were indicated by zone of inhibition around the colony.

### Optimization of media composition, time and pH for antibiotic production

Initially five media AIA^37^, Tryptic soya broth (TSB)^46^, ISP-2^38^, ISP-3^38^, and Starch Casein^47^ were chosen based on their composition to get better antibiotic production^34^. Parameters such as temperature (28 °C), time (7 days), pH (8), shaking speed (180 rpm), and percentage of inocula (1%) were kept constant for all media. The optimal medium was selected for further experiments where the antibiotic production was maximum. With the selected medium we have further tried to scale-up the antibiotic production with a series of time (1–9 days) and pH (5 to 10). In all preliminary experiments only three bacteria (*B. cereus* MTCC 1272, *E. coli* MTCC 1687 and *S. aureus* MTCC 96) and one *S. cerevisiae* were used to determine the inhibitory effect by the crude extract.

### Determination of antimicrobial potency of SM01 culture supernatant

Furthermore, the culture extracts (50 µL) were examined in detail to check the efficacy of the antimicrobial against different test organisms and compared with standard ampicillin and streptomycin antibiotics. The amount of ampicillin for Gram-positive and Gram-negative organisms was 0.1 µg and 5 µg, respectively whereas for streptomycin, it was 5 µg for both organisms.

Antibacterial efficacy has been checked on MH agar seeded with 0.1 ml culture of 0.1 OD (A600 nm) cell of Gram-positive organisms (*S. aureus* MTCC 96, *S. epidermidis* MTCC 3086, *B. cereus* MTCC 1272, *B. stratophericus* MCC 2251, *Enterococcus faecalis* MCC 2041^T^, *Mycobacterium. smegmatis* mc^2^ 155), Gram-negative organisms (*E. coli* MTCC 1687 *P. aeruginosa*, *Klebsiella pneumoniae*, *Salmonella* Typhi), Gram-positive MDR strain including methicillin and vancomycin resistance (*Enteroccocus* sp. 291, *Enteroccocus* sp. and *Staphylococcus haemolyticus*), methicillin-sensitive MDR strain *S. aureus* and Gram-negative MDR strain including methicillin and vancomycin resistance (*Pseudomonas aeruginosa* MV 36846, *Shigella flexneri* IDH 07210, *Salmonella typhimurium* MV 32691, *Klebsiella pneumoniae* MV36808). After 24 hrs (48 hrs for *M. smegmatis* mc^2^ 155) the zone of inhibition has been recorded. The drug-resistant strains used in these studies were isolated from two different hospitals by an independent researcher for different work^5^. The abbreviation in strain name is based on the hospital name and the number is just the serial number of isolated strains.

### Antibiotic sensitivity test by disc diffusion method

To understand the probable antimicrobial categories of the compound produced by SM01, thirty standard antibiotics were tested against SM01. 0.1 ml of the fresh culture of SM01 was spread and antibiotic discs were placed on MH agar plate. This was followed by incubation for 2–3 days at 28 °C. We have used different types of antimicrobial compounds such as (i) cell wall inhibitors: Carbenicillin (10 µg) (CB), Penicillin G (10 U) (P), Cefazolin (30 µg) (CZ), Cefuroxime (30 µg) (CXM), Aztreonam (30 µg) (AT), Amoxocillin + Clavulanic acid (20/10 µg) (AMC/CL), Cefoperazone (75 µg) (CPZ), Vancomycin (30 µg) (VA), Mezlocillin (75 µg) (MZ), Cefoxitin (CE), and Cefepime (30 µg) (CPM); (ii) nucleic acid inhibitors: Norfloxacin (10 µg) (NX), Co-trimoxazole (25 µg) (COT), Ciprofloxacin (5 µg) (CIP), Levofloxacin (5 µg) (LE), Novobiocine (30 µg) (NV), Rifampicin (30 µg) (R), Ofloxacine (5 µg) (OF) and Nitrofurantoin (30 µg) (NIT); (iii) outer membrane inhibitors: Colistin (10 µg) (CL), Polymyxin B (30 µg) (PB); (iv) protein synthesis inhibitors: Oleandomycin (15 µg) (OL), Clindamycin (2 µg) (CD), Lincomycin (15 µg) (L), Tobramycin (10 µg) (TOB), Tetracycline (30 µg) (TE), Chloramphenicol (30 µg) (C), Gentamycin (10 µg) (GEN), Streptomycin (25 µg) (S), Amikacin (30 µg) (AK). Antibiotic sensitivity was observed by measuring the diameter of the zone of inhibition. SM01 was arbitrarily either considered as sensitive (S), intermediate (I) or resistant (R) to an antibiotic. Here we only have compared the extent of sensitivity of antibiotics in a single concentration point (present in the disc). Around the respective antibiotic-disc placed on the solid plate, if *Streptomyces* sp. SM01 failed to form any type of colony it is treated as sensitive; if showed some scattered colonies we designated these as intermediate and if there is no visible inhibition we have marked them as resistant

### Production, extraction, and purification

3-liter AIA medium with 1% inocula of SM01 was incubated at 28 °C for 7 days and medium supernatant was collected by centrifugation for 15 min at 13000 rpm. The active compounds have been extracted after adding an equal volume of ethyl acetate with the medium supernatant. The resulting active organic phase collected and dried by rotary evaporator to concentrate the compound. Crude sample SM1 was first checked by TLC (R_f_: 0.7) with chloroform and ethyl acetate in a 6:1 ratio as a mobile phase solvent. Considering the R_f_ value we further purify the concentrated extract through column chromatography. Upon evaporation of the solvent from the eluted fractions through the rotary evaporator, we got the pure product as a brown solid. The purified product was further tested for its chemical and functional characterization.

### MIC and MBC of bacteria

Minimum inhibitory concentration (MIC) has been estimated in the microplate using MH agar medium. 190 µL MH agar was poured into the well and 10 µL stock solutions (SM1) of the test compound were added into each well. The concentration of the compound used are: set-I (0.025 µg/ml, 0.05 µg/ml, 0.1 µg/ml, 0.2 µg/ml, 0.4 µg/ml, 0.8 µg/ml, 1.6 µg/ml) and set-II (1.6 µg/ml, 3.2 µg/ml, 6.4 µg/ml, 12.8 µg/ml, 25.6 µg/ml, 51.2 µg/ml, 102.4 µg/ml) for different group of test organisms. Bacterial cell, adjusted to 0.1 OD (at 600 nm), from overnight grown culture and further diluted by 1:10 with MH broth. 0.3 µL of this inoculum was spotted on MH agar plate having the desired amount of the test compound. The plates were incubated at 37 °C for 24 hrs and the growth has been observed. MIC was recorded as the lowest concentration of antibiotics where no visible growths were observed.

Minimum bactericidal concentrations (MBC) were analyzed in microplates through broth dilution method^48^. The total MH broth volume taken was 100 µL in each well. 5 µL of test compound (SM1) from different stock solutions, 50 µL of freshly diluted respective cells, and 45 µL of MH broth was added in each well. The final inoculum was 5 × 10^5^ CFU/ml. The cells were incubated in shaking condition overnight at 37 °C. From the overnight grown culture, 10 µL cultures from each well were taken for colony counts and were spread on LA plates and incubated overnight at 37 °C. Plates were observed after 24–48 hrs. MBC was noted as a culture with minimum concentration of SM1 where no colony found on the agar plate.

### MIC and MBC of *Mycobacterium*

*M. smegmatis* mc^2^ 155 were grown in Middlebrook 7H11 broth at 28 °C for 48 hrs. The inoculum suspensions were adjusted to 0.1 OD (at 600 nm) and further diluted by 10 fold. 0.3 µL of this culture were spotted on Middlebrook 7H11 agar in a microplate. In this assay 190 µL medium and 10 µL antibiotics added in each well from different stock solution of antibiotics (1.6 µg/ml, 3.2 µg/ml, 6.4 µg/ml, 12.8 µg/ml, 25.6 µg/ml, 51.2 µg/ml, 102.4 µg/ml). MBC of *M. smegmatis* mc^2^ 155 was analyzed similarly in Middlebrook 7H11 broth

### Cytotoxicity assay

Newly discovered antimicrobials should be assessed for its possible cytotoxicity on the mammalian cell lines. MTT (3-[4,5-dimethylthiazol-2-yl]-2,5-diphenyltetrazolium bromide) assay has been proved to be a reliable and easy process to check the effect of antimicrobials on cell viability. The assay has been found to depend on NAD(P)H-dependent cellular oxidoreductase enzymes present only in viable cells. This enzyme reacts with the MTT dye and produces pink colour compound (formazan) which is soluble in a few organic solvents (*viz*. DMSO). Soluble formazan can be measured by a spectrophotometer at 590 nm wavelength. The extent of color development can be directly correlated with the enzymic activity and indirectly to the number of viable cells. That means higher absorbance indicates the presence of higher viable cells and lower denotes the reduction of cell viability or increase in cell toxicity^49,50^. Cytotoxicity of the isolated antibiotic has been analysed through MTT assay on A549 (ATCC No CCL-185) cancer cell line. The cell line has been maintained in Dulbecco Modified Eagle Medium (DMEM) supplemented with 10% fetal bovine serum with 1% Penicillin-Streptomycin at 37 °C in an incubator with 5% CO_2_. Cells were harvested with trypsin and centrifuge 1200 rpm for 10 min. After decanting the soup, fresh medium was added to the pellet to adjust 10^4^ cells in 100 µL medium. All tests have been performed in triplicate. Plates were incubated overnight at the above mentioned condition. Overnight grown cells were treated with antibiotics in different concentrations followed by further overnight incubation under the same condition. Following incubation 20 µL MTT reagents (5 mg/ml) were added and incubated at shaking condition for 4 hrs. The soups were removed carefully and 150 µL MTT solvent (DMSO) was added to each well to dissolve the formazan. After 1 hr the absorbances were measured at 590 nm.

### Structural elucidation of the newly isolated compound

All the solvents (CHCl_3_ and ethyl acetate) were distilled and dried following the standard procedures^51^. ^1^H and ^13^C NMR spectra were recorded on a 400 MHz instrument. For NMR spectra and NMR titration CDCl_3_ was used as solvent using TMS as an internal standard. Chemical shifts have been expressed in δ ppm units and ^1^H–^1^H and ^1^H–C coupling constants in Hz. The mass spectrometry (HRMS) was carried out using a micromass Q-TOF MicroTM instrument by using methanol as a solvent. Fluorescence spectra were recorded on a spectrophotometer. FTIR spectra were recorded as KBr pellets using a spectrophotometer. UV spectra were recorded on a spectrophotometer. Elemental analysis of the compound was carried out on CHNS/O analyzer. The following abbreviations have been used to describe spin multiplicities in ^1^H NMR spectra: s = singlet; d = doublet; t = triplet; m = multiplet.

## Results

### Isolation and identification of SM01

**Figure 1:**
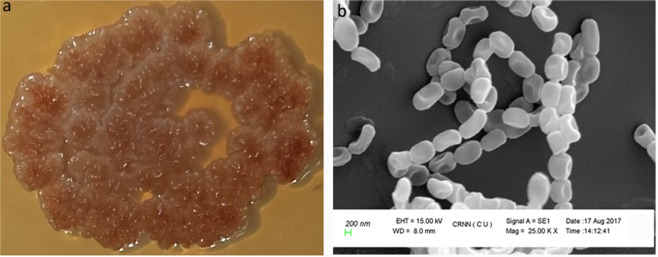
Morphology of isolated SM01. (**a**) appearance of SM01 colony in ISP-2 agar medium (**b**) scanning electron micrograph of SM01.

SM01 was isolated from an unexplored region of Rangreth, Himalayan Kashmir, India, in AIA medium. SM01 identified as a *Streptomyces* (Fig. 1a), the most abundant actinomycetes in soil, and it has a massive contribution to drug industry^34,52^. The colour of aerial and substrate mycelium is tan in appearance if grown in ISP-2 and AIA medium. It does not produce any diffused pigment if grown in mentioned solid medium however it can produce deep brown pigment in submerged condition while grown in AIA liquid medium. The colony of SM01 has found to be filamentous, dull, umbonate, rough, and translucent. Scanning electron micrograph shows that the aerial mycelium produces spore chain and the spore surfaces are smooth (Fig. 1b). The 16 S rDNA gene sequence of SM01 is having 99.30% similarity with *Streptomyces levis* NBRC 15423. Phylogenetic analysis by NJ, ML, and MP tree shows that SM01 belong to separate cluster which might indicate that SM01 is a new species (see Supplementary Fig. S1).

### Antimicrobial activity of SM1

From preliminary screening it has been found that SM1 inhibits the growth of both Gram-positive and Gram-negative organisms however, the growth impairment of yeast is very less. Upon optimization, the highest antibiotic production by SM01 has been found in AIA medium among 5 different medium tested (Table 1) considering the wet weight of SM01 cell. Interestingly among the five tested media for antibiotic production-optimization study, all media (except AIA) i,e starch casein, ISP-2, ISP-2 and TSB support larger quantity [wet weight (g)] of cell mass but showed smaller zone of inhibition against tested bacteria. We have observed that the growth and antibiotic production are not proportional as some medium support larger cell mass without enhancement in antibiotic production. Although in starch casein and AIA medium the strain SM01 produces an almost equal quantity of antibiotic (as measured in terms of antimicrobial activity), after normalizing (each gram of cell) the cell mass it has been found that in starch casein medium the amount of antibiotic production is lowest. Interestingly it has been found that strain SM01 is sensitive against most of the tested antibiotics, weakly sensitive against seven antibiotics and resistance against none (see Supplementary Table S1). This indicates that SM01 should not be the producer strain of any of these tested antibiotics, as in general the strain become resistant against the self-produced antibiotics. Thus we predict that the SM1 produced by strain SM01 is different from these tested antibiotics and might have the possibility to be a new antimicrobial agent.

**Table 1:** Medium optimization for picolinamycin production.

Medium	Wet weight (g)/100 ml	*S. aureus*	Zone of inhibition (mm)	*E. coli*
			*B. cereus*	
AIA	2.135	26	21	11
ISP-2	2.71	18	14	14
ISP-3	4.53	23	19	10
Starch casein	5.43	25	21	10
Tryptic soya broth	2.59	24	11	05

**Figure 2:**
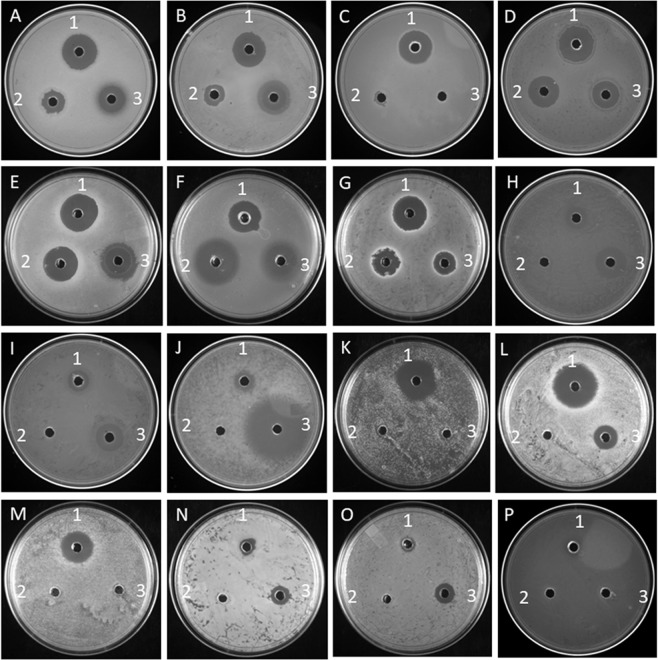
Antimicrobial efficacy of crude SM1. Efficacy detected by zone of inhibition against different organisms using SM1, ampicillin, streptomycin [A = *S. aureus* MTCC 96, B = *S. epidermidis* MTCC 3086, C = *B. cereus* MTCC 1272, D = *B. stratosphericus* MCC 2251, E = *E. faecalis*, F = *S*. Typhi, G = *K. pneumoniae*, H = *E. coli* MTCC 1687, I = *P. aeruginosa*, J = *M. smegmatis* mc^2^ 155, K = *S. haemolyticus* (MDR)., L = *S. aureus* (MDR), M = *Enterococcus* sp, N = *Enterococcus* sp. 291, O = *P. aeruginosa* MV36846 (MDR), P = *S. flexneri* IDH 07210 (MDR)]; 1 = 50 µl medium supernatant of SM01, 2 = Ampicillin (5 µg for Gram-negative bacteria, 0.1 µg/ml for Gram-positive bacteria), 3 = Streptomycin (5 µg).

It has also been found that the cell-free culture medium of SM01, grown in AIA medium with pH 8 for 7 days (see Supplementary Table S2 and Table S3), showed maximum zone of inhibition against tested organisms. In a qualitative assay, it has been found that 50 µL of culture supernatant showed a larger zone of inhibition against Gram-positive organisms compared to standard ampicillin and streptomycin (5 µg) (Fig. 2). In our study, among the total eight MDR strain tested, we have found that the crude culture supernatant of SM01 was able to show inhibitory action against six MDR strains (except *K. pneumoniae* MV36808 and *S. typhimurium* MV32691). The MDR strains of *S. haemolyticus*, *Enterococcus* sp, and *S. flexneri* IDH 07210 are resistant to ampicillin and streptomycin but can be inhibited by the newly isolated compound SM1. Similarly MDR strains of *S. aureus*, *Enterococcus* sp 291, *P. aeruginosa* MV 36846 are found to be resistant against ampicillin (although sensitive by streptomycin) but compound SM1 can kill these organisms effectively. Also in quantitative assay the purified compound SM1 is effective against nearly all tested MDR strains (except *K. pneumoniae* MV36808 and *S. typhimurium* MV32691) (see Supplementary Fig. S2). MIC of the compound has been found as 0.01 µg/ml for non-MDR Gram-positive organisms (except *M. smegmatis* mc^2^ 155) and from 0.02 to 5.12 µg/ml for non-MDR Gram-negative organisms. For MDR and MDR including methicillin resistance organisms, the MIC value of the purified antimicrobials is in the range of 0.04 to 5.12 µg/ml. MIC value for *M. smegmatis* is 10.24 µg/ml (Table 2). Though the crude culture-supernatant showed a sub-inhibitory effect against *S. cerevisiae* but the purified compound has been failed to show any growth impairment. The inhibitory action of the crude culture-supernatant might be for other compounds mixed with SM1 in the crude culture supernatant.

**Table 2:** MIC and MBC of picolinamycin.

Test organisms	MIC (µg/ml)	MBC (µg/ml)
*S. aureus* MTCC 96	0.01	1.28
*S. epidermidis* MTCC 3086	0.01	>50
*B. cereus* MTCC 1272	0.01	5.12
*B. stratosphericus* MCC 2251	0.01	20.48
*E. faecalis* MCC 2041^T^	0.01	50
*S. Typhi*	0.08	25
*K. pneumoniae*	0.02	>50
*E. coli* MTCC 1687	2.56	50
*P. aeruginosa*	5.12	20.48
*M. smegmatis* mc^2^ 155	10.24	>50
**MDR strains**		
*S. haemolyticus*	0.08	>50
*S. aureus*	0.08	5.12
*Enterococcus* sp.	0.04	1.28
*Enterococcus* sp.291	5.12	>50
*P. aeruginosa* MV36846	2.56	>50
*S. flexneri* IDH07210	2.56	50

### Structural elucidation of compound SM1

**Figure 3:**
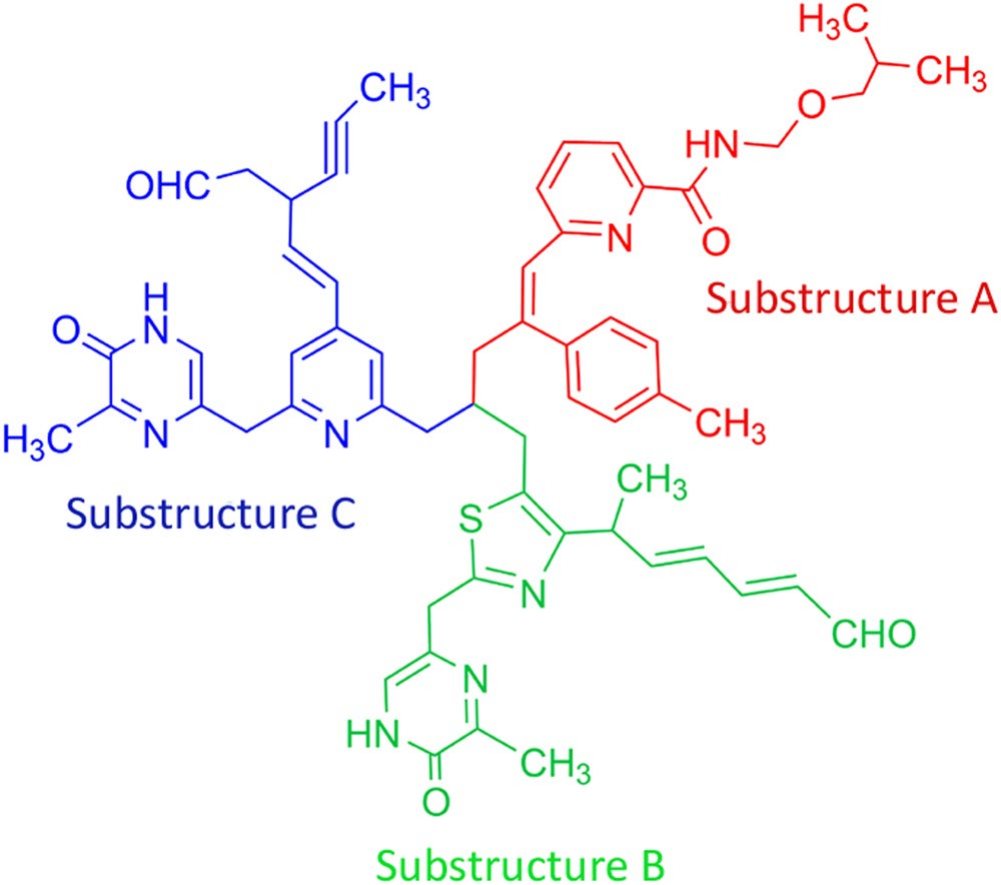
The total structure of picolinamycin with Substructure A (in red), B (in green) and C (in blue).

3 liters of SM01 culture was centrifuged and the cell pellet was discarded. The cell-free supernatant was mixed with an equal volume of ethyl acetate and the collected organic fraction was dried in vacuum. The crude mixture was first checked in a thin layer chromatography (TLC) plate (Silica gel 60 F_254_) using chloroform and ethyl acetate (ratio 6:1, v/v), R_f_ = 0.70. Then flush column chromatography was performed in the Silica gel bed (60-120 mesh) with the same eluent ratio of the solvents. Structural characterization was performed by CHN analysis, mass spectrometry, UV-Visible and fluorescence spectroscopy, IR, ^1^HNMR and ^13^CNMR. The mass spectrum of SM1 showed a base peak [M + K + 2H]^+^ at m/z 1053.4244 (see Supplementary Fig. S3) (calculated mass: 1012.4670). The molecular formula has been determined to be C_59_H_64_N_8_O_6_S (Fig. 3) based on HRMS analysis data by considering the number of protons and carbons from the NMR spectrum (Table 3). S is surely present in SM1 as we have found positive results for the lassaigne test. The compound showed absorption maxima at 252 and 318 nm and emission maxima at 385 nm (see Supplementary Table S4, Figs. S4, and S5). Absorption at 2962, 1736, 1259, 3393 and 1500 cm^-1^ in the IR spectrum suggests the presence of amide, carboxy ether, ethelynic, acetylinic carbon and phenyl groups (see Supplementary Fig. S6).

**Table 3:** ^1^H and ^13^C NMR spectral data of picolinamycin in CDCl_3_.

Position	δ_H,_ J(Hz)	δ_c_	Position	δ_H,_ J(Hz)	δ_c_	
1	0.85(d, 3H)	18.79	32	—	46.59	
2	1.39(m 1H)	29.68	33	9.60(d, 1H)	173.42	
3		0.85(d, 3H)	18.79	34	7.81(s, 1H)	130.99
4	1.36(d, 2H)	62.73	35	—	164.07	
5	2.99(d, 2H)	62.73	36	0.87(s, 2H)	32.24	
6	—	171.13	37	—	129.62	
7	—	130.99	38	7.03(d, 1H)	132.0	
8	8.62(d, 1H)	129.23	39	—	170.27	
9	8.13(d, 1H)	140.11	40	—	168.85	
10	8.62(d, 1H)	129.23	41	3.11(s,3H)	18.79	
11	—	163.95	42	2.99(d, 2H)	46.27	
12	4.89(s, 1H)	129.62	43	—	129.65	
13	—	143.63	44	—	144.11	
14	—	140.81	45	4.64(m,1H)	32.24	
15	7.81(d, 1H)	129.65	46	1.06(d, 1H)	20.39	
16	7.81(d, 2H)	131.09	47	4.82(d, 1H)	132.08	
17	—	129.36	48	5.11(d, 1H)	142.30	
18	7.91(d, 2H)	131.09	49	4.75(m,1H)	167.62	
19	7.85(d, 1H)	129.65	50	4.94(d, 1H)	142.30	
20	2.99(d, 2H)	46.27	51	9.60(d, 1H)	173.42	
21	1.06(m,1H)	32.24	52	—	167.40	
22	3.01(d, 2H)	46.27	53	1.39(s, 2H)	129.62	
23	—	163.95	54	—	129.23	
24	7.81(s, 1H)	132.08	55	—	164.07	
25	—	142.30	56	2.078(s,3H)	18.05	
26	4.98(d, 1H)	129.62	57	—	168.85	
27	4.96(d, 1H)	129.65	58	7.89(s, 1H)	131.0	
28	4.64(m,1H)	20.39	NH_a_	6.09(d, 1H)		
29	—	64.98	NH_b_	6.91(d, 1H)		
30	—	64.24	NH_c_	7.03(d, 1H)		
31	0.896(s,3H)	14.11				

The detailed NMR studies (^1^H NMR, ^13^C NMR, DEPT-135, COSY and HMBC) were performed to establish the structure of SM1 (see Supplementary Figs. S7–S11). The ^13^C NMR spectrum showed 58 signals that were assigned to 6 methyl, 4 methine, 8 methylene and 20 quaternary carbons along with 18 olefinic carbons by heteronuclear multiple quantum coherence (see Supplementary Fig. S7). The total structure of SM1 is consists of substructure A, B, and C (Fig. 3). SM1 contains a central picolinamide moiety. As this antibiotic compound produced by *Streptomyces* sp and having a picolinamide moiety we name this as picolinamycin.

**Figure 4:**
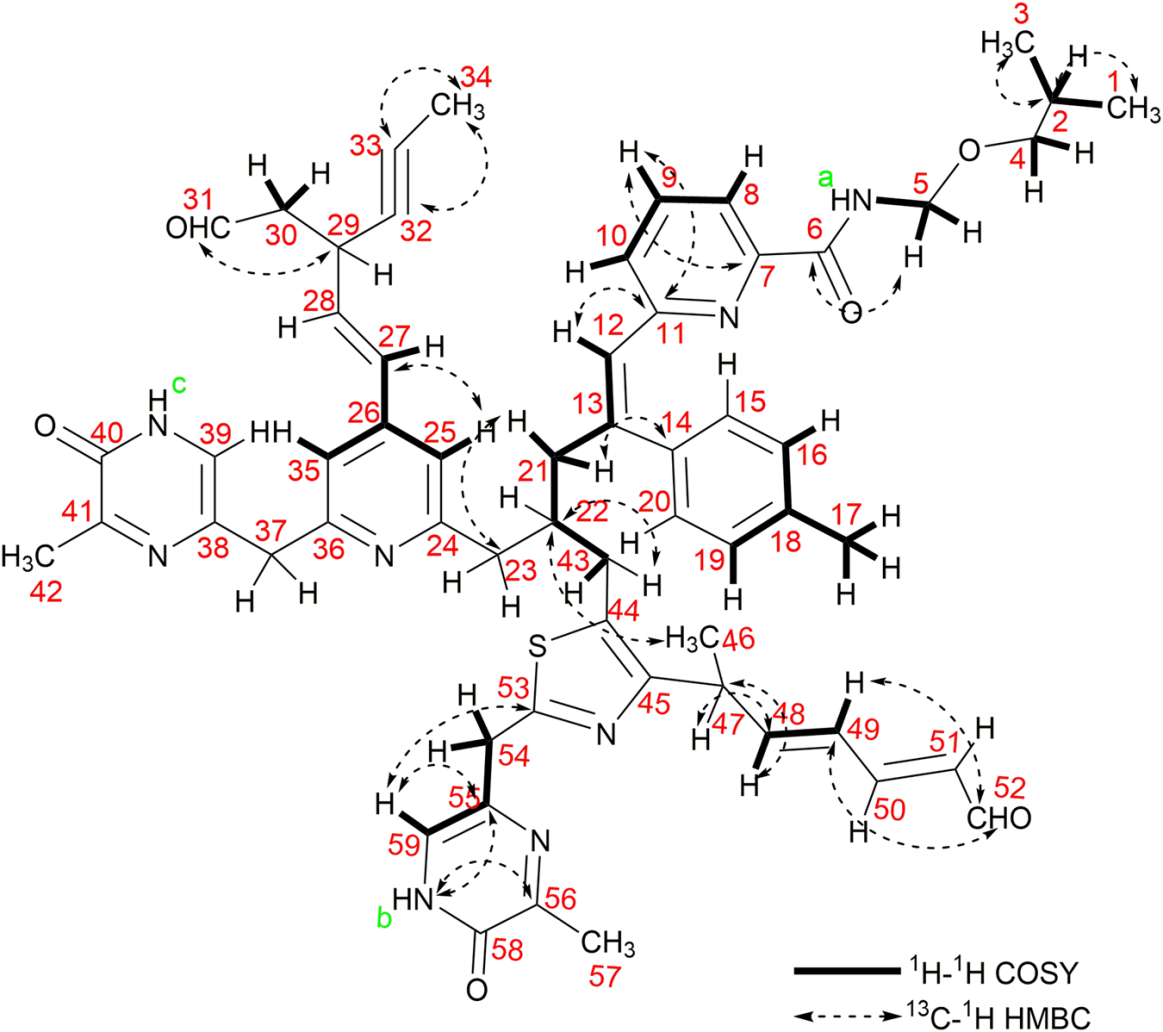
2D NMR (HMBC) correlation of picolinamycin.

Substructure A:

Substructure A is one hand of compound picolinamycin (Fig. 3). It contains a pyridine ring along with a benzene ring. The *ortho*-coupled aromatic protons at δ 6.91, δ 7.03 (H8, H10), and δ 8.13 (H9) were connected by the COSY spectrum (Fig. S10). H9 showed a long range correlation to δ 130.99 (C7) and δ 163.95 (C11), and a long range coupling from H9 with C7 and C11 was observed. These data revealed the presence of a pyridine ring (Fig. 4). The methyl proton signal at δ 1.36 (H4), δ 2.99 (H5) showed long range coupling to the oxygenated methyl carbon at δ 61.97, δ 62.73 (C4, C5). Moreover long range correlation from the methyl proton signal at δ 0.85 (C1, C3) to δ 29.68 (C2) was observed. The CH_2_ proton δ 2.99 (H5) was coupled to an amide proton at δ 6.09 (NH_a_) and showed a long range correlation to δ 171.13 (C6). Furthermore, long range coupling from the proton signal of the pyridine unit at δ 6.09 (NH_a_) to the quaternary carbon of methylbenzene at δ 140.81 (C14) was observed. In COSY spectrum, vicinal coupling signals were detected between the methyl protons at δ 0.85 (H1, H3) and the methane proton at δ 1.39 (H2) and aromatic protons H16 (δ 6.48) and H19 (δ 7.91) were connected with the methyl protons at δ 3.11 (H17). The presence of long range coupling from the proton signal of the aliphatic chain δ 4.89 (H12), δ 2.99 (H21) to the quaternary carbon of pyridine (δ 163.95, C11) and methylbenzene (δ 140.81, C14) indicated the presence of substructure A.

Substructure B:

Substructure B contains a pyrimidine ring and a thiazole ring (Fig. 3). The aromatic proton (H43) at δ 2.99, δ 4.89 (H12) and δ 2.99 (H21) are connected by the COSY spectrum. H59 showed a long range correlation to δ 129.23 (C55) and δ 167.40 (C53). An analysis of HMBC spectroscopic data provides further structural information on substructure B (see Supplementary Fig. S11). The cross peak from δ 8.62 (NH_b_) to δ 129.23 (C55) and δ 164.07 (C56) from H_3_-57 (δ 2.078) to C56 supports the partial structure of the center ring. Furthermore, long range coupling from proton signal of the pyrimidine unit at δ 7.89 (H59) to the quaternary carbon of the thiazole unit at δ 167.40 (C53) and from δ 132.08 (C48) to δ 4.64 (H47) has been observed. The aromatic proton H59 (δ 7.89) is connected with methylenic protons H54 (δ 1.39) by COSY spectrum. The presence of long range couplings from the proton signal of the thiazole unit at δ 32.24 (C47) to δ 1.06 (H46), δ 4.82 (H48) along with the olefinic protons at δ 5.11 (H49) δ 4.94 (H51) to the aldehydic carbon δ 173.42 (C52) indicates the presence of substructure B.

Substructure C:

Substructure C contains a pyridine ring and a pyrimidine moiety attached with a methane (CH_2_) group (Fig. 3). The ortho coupled aromatic protons at δ 7.81 (H25, 35) and δ 4.98 (H27) are connected by the COSY spectrum. From the HMBC spectroscopic analysis the presence of pyrimidine moiety (in substructure C) attached with a pyridine via CH_2_ group has also been determined (same as substructure B).

A long range coupling from the proton signal of the pyridine unit at δ 7.81 (H25, 35) to the methylene carbon δ 129.62 (C27) and δ 129.65 (C28) was observed. The presence of long range coupling from the proton signal of olefinic protons δ 4.98 (H27) δ 4.96 (H28) to acetylinic carbon δ 64.98 (C32), δ 64.24 (C33) along with the aldehydic carbon δ 173.42 (C31) has also been revealed. The cross coupling of terminal methyl group δ 0.896 (H_3_ 34) to C33 and C32 indicates the presence of substructure C.

In HMBC spectra, it has been observed that there is three-bond cross coupling between H21 (δ 2.99)-C23 (δ 46.27) and two and five-bond cross coupling between C22 (δ 32.24)- H43 (δ 2.99) and C22 (δ 32.24)- H46 (δ 1.06) respectively, which evidence that the three substructures are connected through C22 carbon as shown in Fig. 4.

## Discussion

In our previous study we have shown that the soil sample collected from Kashmir regions are the great reservoirs for actinomycetes including *Streptomyces* sp with great industrial potentials^34^. One among these isolates has been identified as *Streptomyces* sp SM01. The 16 S rDNA sequence-based phylogenetic analysis along with morphological and biochemical analysis has been indicated strain SM01 as a species of *Streptomyces*. We have performed small scale fermentation of SM01 in different media with different temperatures, pH and incubation time to optimize the production of the compound SM1. Upon optimization, the AIA medium has been considered as the most favorable medium for the highest amount of antibiotic production. All the tested media contain different carbon and nitrogen sources; as yeast extract, malt extract, dextrose in ISP-2; oat meal in ISP-3; starch, casein in starch casein medium; casein, peptic digest of soyabean meal, dextrose in TSB; and sodium propionate, sodium caseinate, L-asparagine and glycerol in AIA medium. Considering these nutrients factors, one can predict that the components of AIA like sodium propionate, L-asparagine and glycerol might have roles for higher yield of the antimicrobial compound which has also been supported partially by Ilic *et al*.^53^. The overall yield of the purified compound SM1 is 4 mg per liter culture. The total structure of SM1 is consists of substructure A, B and C with a molecular weight of 1013 dalton (Fig. 3). SM1 contains a central picolinamide moiety. As this antibiotic compound produced by *Streptomyces* sp and having a central picolinamide moiety, we name this as picolinamycin. Upon searching the chemical databank, we have concluded that picolinamycin is a novel compound of bio-origin.

**Figure 5:**
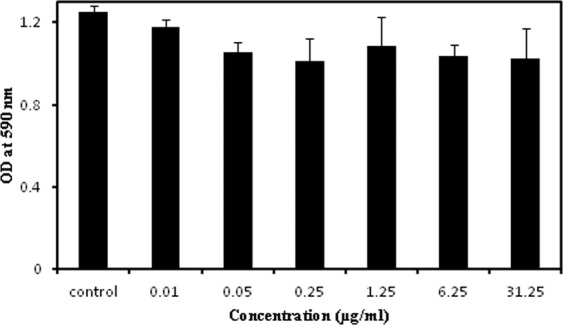
Cytotoxicity assay of picolinamycin against A549 (ATCC No CCL-185) human cell line. Various concentration of picolinamycin was added in respective well having cells. Each concentration point was taken in triplicate.

Nosocomial pathogens having multidrug resistance (MDR) or pandemic drug resistance (PDR) traits usually represent a paradigm in pathogenesis and responsible for life-threatening diseases in human^54,55^. The non-judicial usage of available antibiotics remains as the main reason for the crisis of having significant and effective antibiotic against resistance pathogens^8–10^. The biggest challenge in the present-day is to find suitable antibiotics with a unique mode of action to combat the MDR and PDR strains. It has been found that the purified compound picolinamycin is highly effective against many hospital-borne deadly pathogens having vancomycin and oxacillin resistance phenotype. Among the hospital-acquired bacteria, coagulase-negative (CoNS) *Staphylococci* are one of the leading causes of nosocomial infection. Currently *S. haemolyticus* has also found to be resistant to too many existing antibiotics^56,57^ including methicillin and vancomycin. We have found that the newly characterized picolinamycin is effective against MDR *S. haemolyticus* (MIC 0.08 µg/ml). Another MDR bacteria (especially resistant to vancomycin and methicillin), *Enterococcus* is the second leading (after *S. aureus*) nosocomial pathogen^58^ and sensitive to picolinamycin (MIC 0.04 µg/ml). Furthermore, the growth of *S. aureus* and its methicilin-sensitive but vancomycin-resistant MDR strains are also inhibited (MIC 0.01 µg/ml and 0.08 µg/ml, respectively) by picolinamycin. Similarly, Gram-negative bacteria like *Pseudomonas* which is responsible for about 20% nosocomial infection in US^59^ also get prevented by this antibiotic although MIC value (MIC 2.56 µg/ml) higher compared to Gram-positive organisms. Picolinamycin can also be used to inhibit the growth of *M*. *smegmatis* mc^2^ 155 (MIC 10.24 µg/ml) which indicates that it might similarly be effective against the tuberculous bacilli. Apart from MDR strains picolinamycin remains remarkably effective against most ESKAPE strains (*Enterococcus. faecium, Staphylococcus aureus, Klebsiella pneumoniae, Acinetobacter baumanni, Pseudomonas aeruginosa, and Enterobacter* species)^6^ which are indigenous group of nosocomial organisms. The microbicidal activity of picolinamycin has also been tested on various pathogenic organisms. It is generally said that if minimum bactericidal concentration (MBC) is less than 4-times of the MIC or equals to MIC then the drug is known to be bactericidal^60^. We have noticed that the MBC value of picolinamycin is found to be much higher than MIC against all organisms, indicating its bacteriostatic properties. Picolinamycin up to a concentration of 31.25 µg/ml could not exert any cytotoxicity against the tested human A549 cell line (Fig. 5). This predicts that the antibiotic works against the bacterial pathogens in a unique way where the target is not overlapped with the eukaryotic organisms, emphasizing its properties as an ideal antibiotic. More precisely *Streptomyces* sp SM01 is a novel antibiotic producer that was isolated from Indian soil. This antibiotic has successfully characterized through spectroscopy methods and denoted as picolinamycin. It is active against both drug resistance and sensitive Gram-positive and Gram-negative bacteria. Hence picolinamycin has the potentiality to use industrially as next-generation drug of choice for MDR strains.

## Supplementary information

Dataset 1.
