# Supplementary material for: Streptomyces sp SM01 isolated from Indian soil produces a novel antibiotic picolinamycin effective against multi drug resistant bacterial strains
Source: Sci Rep. 2020 Jun 22;10:10092. doi: 10.1038/s41598-020-66984-w (PMC7308314; doi:10.1038/s41598-020-66984-w)
Supplement: Supplementary file 1 — Dataset 1. [file 41598_2020_66984_MOESM1_ESM.pdf]

***Streptomyces* sp SM01 isolated from Indian soil produces a novel antibiotic picolinamycin effective against multi drug resistant bacterial strains.**

Author: Pulak Kumar Maiti[1#], Sujoy Das[2#], Prithidipa Sahoo[2*], Sukhendu Mandal[1*]

Affiliations:

[1]Laboratory of Molecular Bacteriology, Department of Microbiology, University of Calcutta, 35, Ballygunge Circular Road, Kolkata, 700019, India

[2]Molecular Recognition Laboratory, Department of Chemistry, Visva-Bharati University, Siksha Bhavana, Santiniketan, Birbhum, West Bengal-731235, India

[#] These two authors contributed equally

* To whom correspondence should be addressed: sukhendu1@hotmail.com

Corresponding Author: Sukhendu Mandal

Address: Laboratory of Molecular Bacteriology, Department of Microbiology, University of Calcutta, 35, Ballygunge Circular Road, Kolkata, 700019, India.

Email: sukhendu1@hotmail.com

Co-corresponding Author: Prithidipa Sahoo

Address: Molecular Recognition Laboratory, Department of Chemistry, Visva-Bharati University, Siksha Bhavana, Santiniketan, Birbhum, West Bengal-731235, India.

Email: prithidipa@hotmail.com

Table S1: Antibiotic sensitivity of *Streptomyces sp.* SM01 against standard antibiotics disc

Antibiotic disc	Interpretation
Carbenicillin	S
Penicillin G	I
Cefazolin	I
Cefuroxime	S
Aztreonam	I
Amoxocillin/Clavulanic acid	S
Cefoperazone	S
Vancomycin	S
Mezlocillin	S
Cefoxitin	S
Cefepime	I
Norfloxacin	S
Co-trimoxazole	I
Ciprofloxacin	S
Levofloxacin	S
Novobiocine	S
Rifampicin	S
Ofloxacine	S
Nitrofurantoin	S
Colistin	S
Polymyxin B	S
Oleandomycin	S
Clindamycin	S
Lincomycin	S
Tobramycin	S
Tetracycline	I
Chloramphenicol	S
Gentamycin	S
Streptomycin	S
Amikacin	I

Table S2: pH optimization for SM1 compound production

pH	Zone of inhibition (mm)		
	S. aureus	*B. cereus*	*E. coli*
5	14.5	19	-
6	15	20	-
7	22	25	15
8	28	26	12
9	19	25	10
10	17	22	10-

Table S3: Time optimization for SM1 compound production

Day	Zone of inhibition (mm)		
	S. aureus	*B. cereus*	*E. coli*
1	-	-	-
2	11	03	-
3	18	21	-
4	20	18	-
5	20	18	-
6	20	18	12.5
7	22	26	12
8	21	20	12
9	21	23.5	12

Table S4: Physicochemical properties of SM-1

Appearance	Amorphous brown solid
Molecular Formula	$C_{59}H_{64}N_8O_6S$
Molecular weight	1013.2710
HRMS m/z	$(M+K+2H)^+$
Calcd	1012.4670
Found	1053.4244
UV (Acetonitrile)	252, 318
IR (KBr) cm^{-1}	2962, 1736, 1259, 3393, 1500

Figure S1

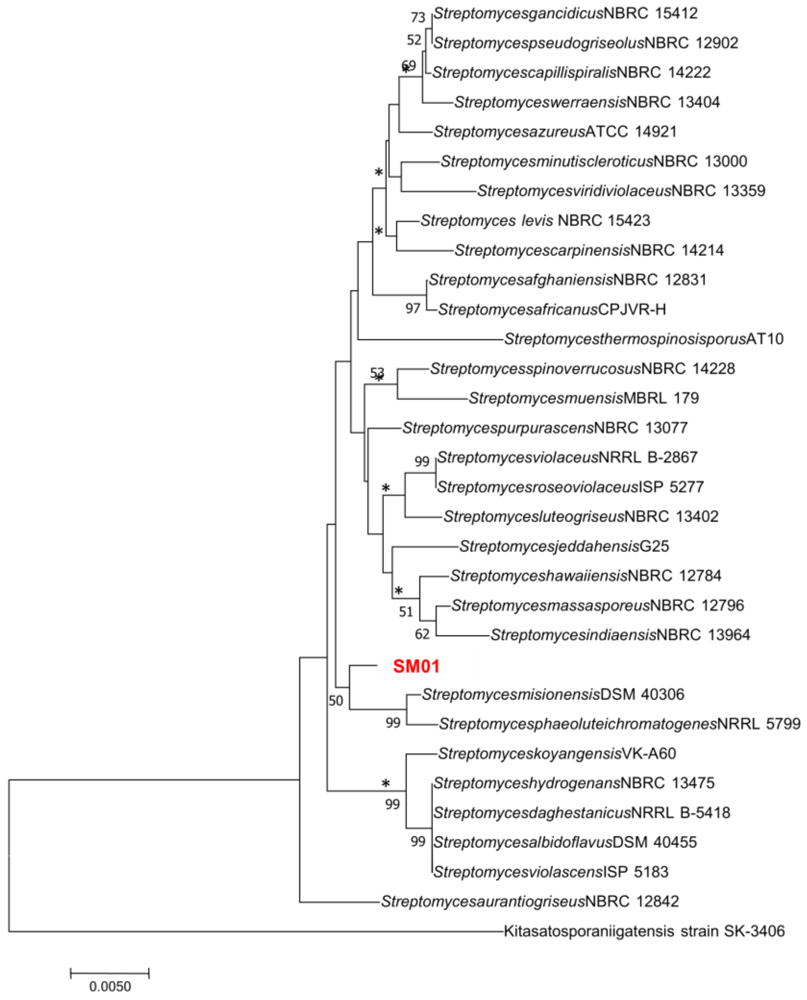

Figure S1: Phylogenetic position of SM01 (Neighbour joining tree based on 16S rDNA linear sequence. Only >50% bootstrap values are shown at branch node from 1000 replicants. * indicates cluster showed in both ML and MP tree)

Figure S2

(a)

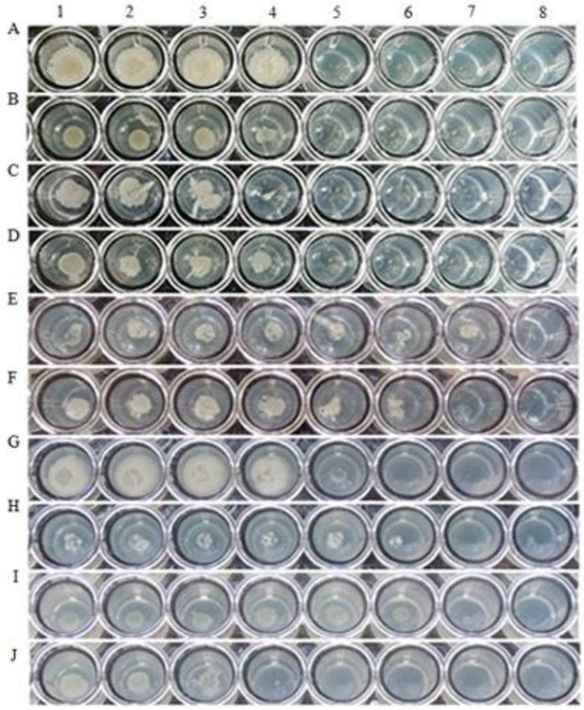

(b)

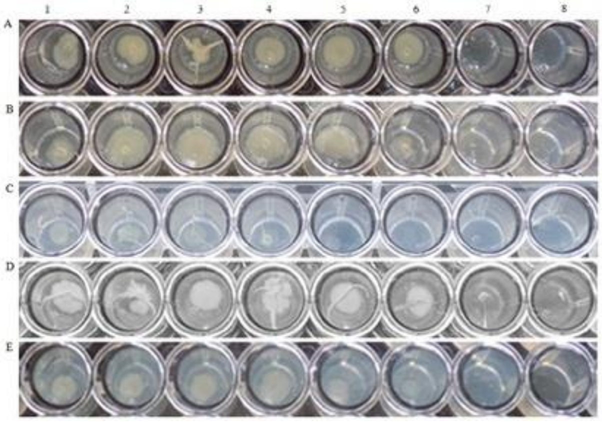

(c)

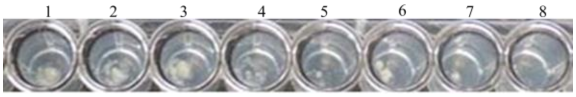

Figure S2: Antimicrobial efficacy of purified picolinamycin (a) MIC of purified picolinamycin (with set-I concentration range) against different organisms [picolinamycin concentrations used are: 1=control without antibiotic, 2=0.00125 µg, 3=0.0025 µg, 4=0.005 µg, 5=0.01 µg, 6=0.02 µg, 7=0.04 µg, 8=0.08 µg; organisms tested are: A=*B. cereus* MTCC 1272, B=*B. stratosphericus* MCC 2251, C=*S. aureus* MTCC 96, D=*S. epidermidis* MTCC 3086, E=*S. haemolyticus* (MDR), F= *S. aureus* (MDR), G= *Klebsiellia sp.*, H=*Enterococcus* (MDR), I= *S. typhi*, J= *E. faecalis* MCC 2041^{T}]. (b) MIC of purified picolinamycin(with set-II concentration range) against different organisms [picolinamycin concentrations used are: 1=control without antibiotic, 2=0.08 µg, 3=0.16 µg, 4=0.32 µg, 5=0.64 µg, 6=1.28 µg, 7=2.56 µg, 8=5.12 µg; organisms tested are: A= *Pseudomonas aeruginosa* MV36846 (MDR), B=*E. coli* 1687, C=*P. aeruginosa*., D=*Enterococcus sp.* 291, E= *S. flexneri* IDH07210]. (c) MIC of purified picolinamycin against *M. smegmatis* [concentrations used are: 1=control without antibiotic, 2=0.16 µg, 3=0.32 µg, 4=0.64 µg, 5=1.28 µg, 6=2.56 µg, 7=5.12 µg 8= 10.24 µg]

Figure S3

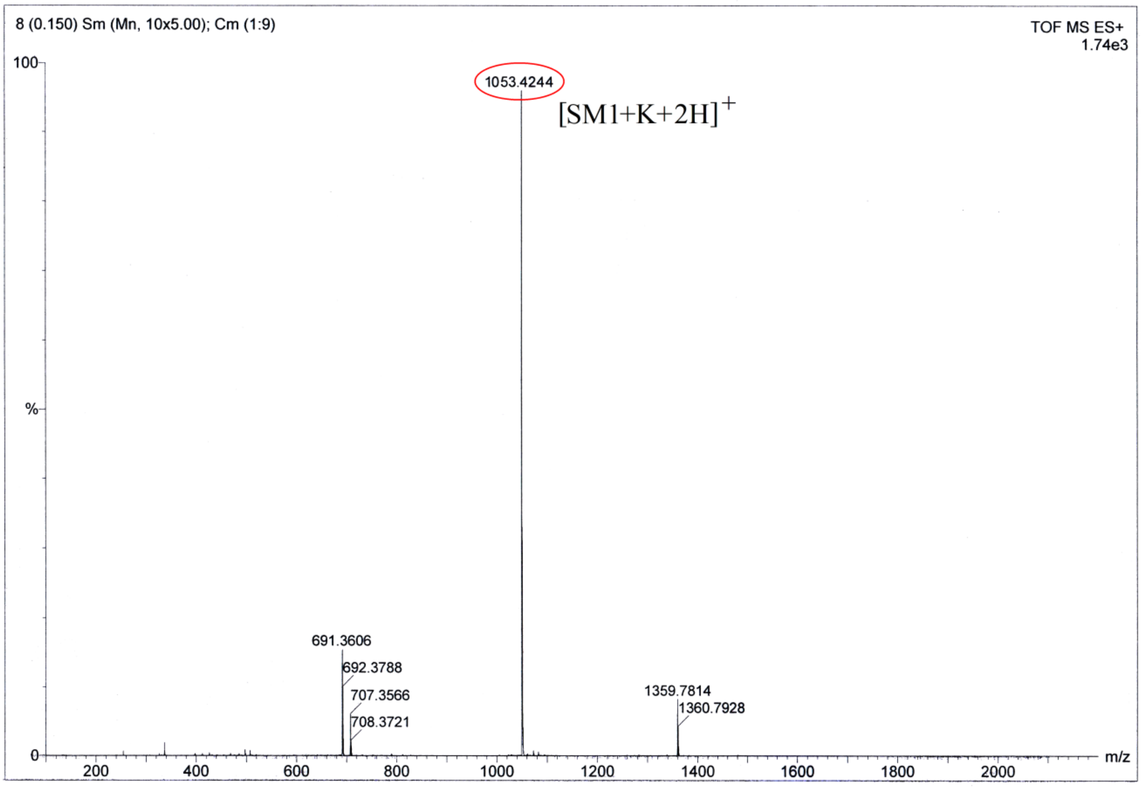

Figure S3: HRMS spectra of picolinamycin

Figure S4

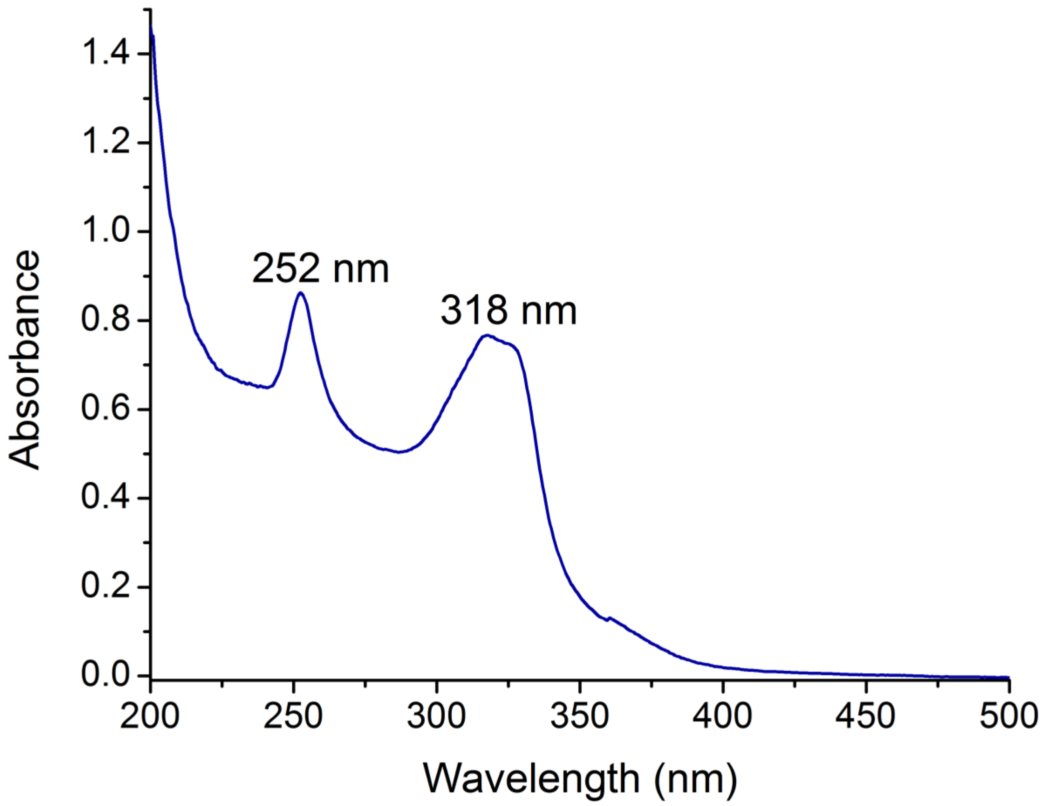

Figure S4: Absorbance plot of picolinamycin

Figure S5

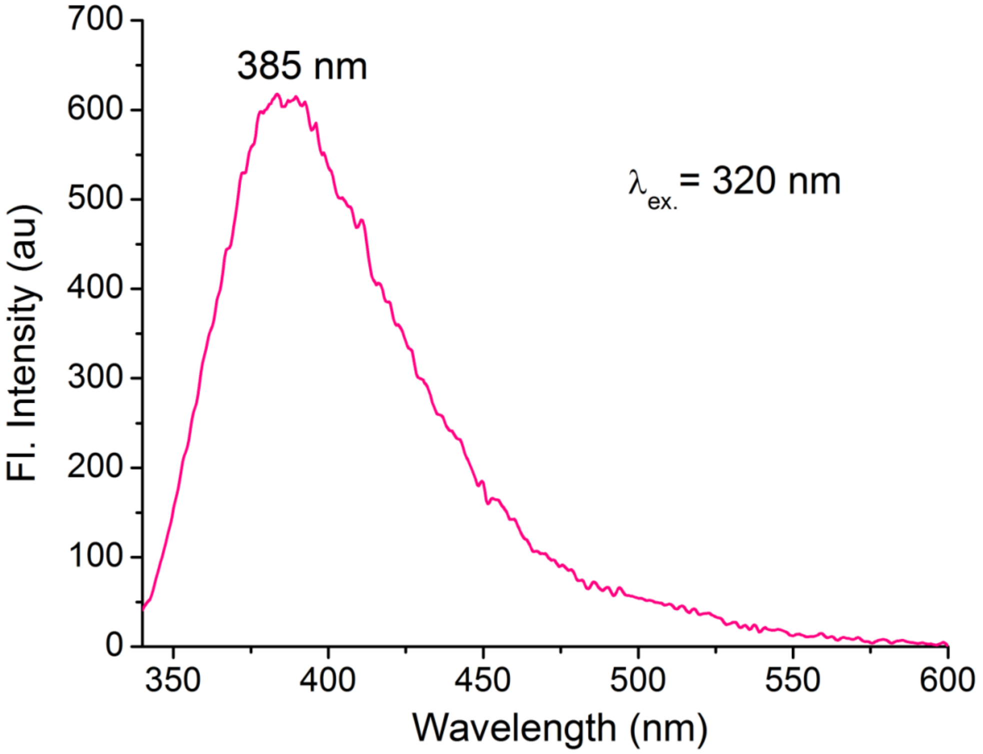

Figure S5: Fluorescence plot of picolinamycin

Figure S6

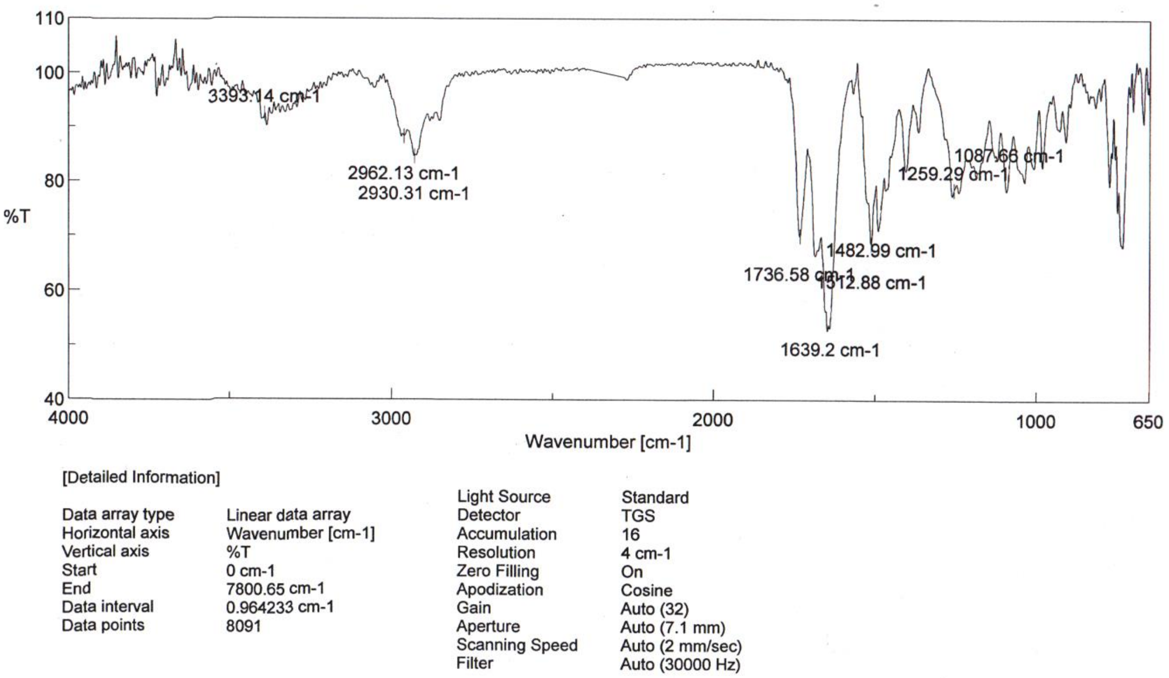

Figure S6: FTIR spectra of picolinamycin

Figure S7

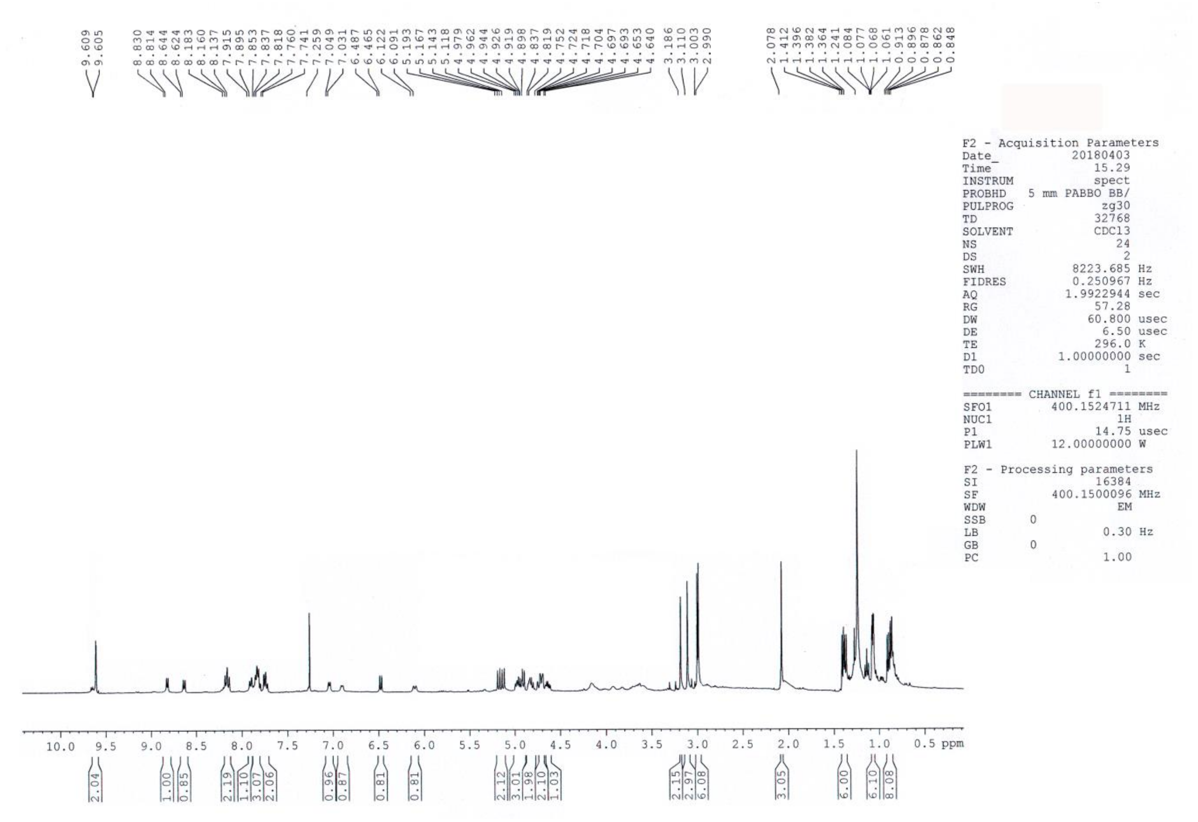

Figure S7: ^{1}H NMR of picolinamycin in $CDCl_3$

Figure S8

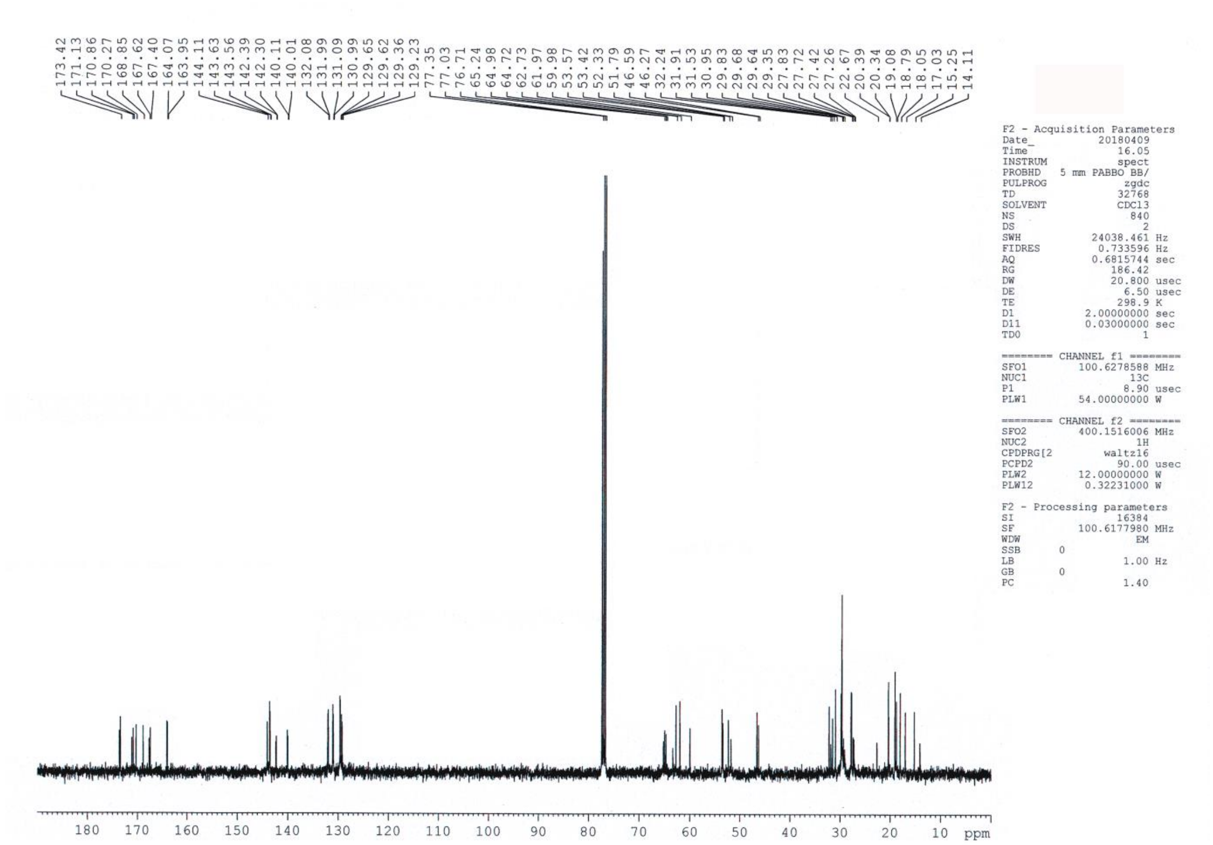

Figure S8: ^{13}C NMR of picolinamycin in $CDCl_3$

Figure S9

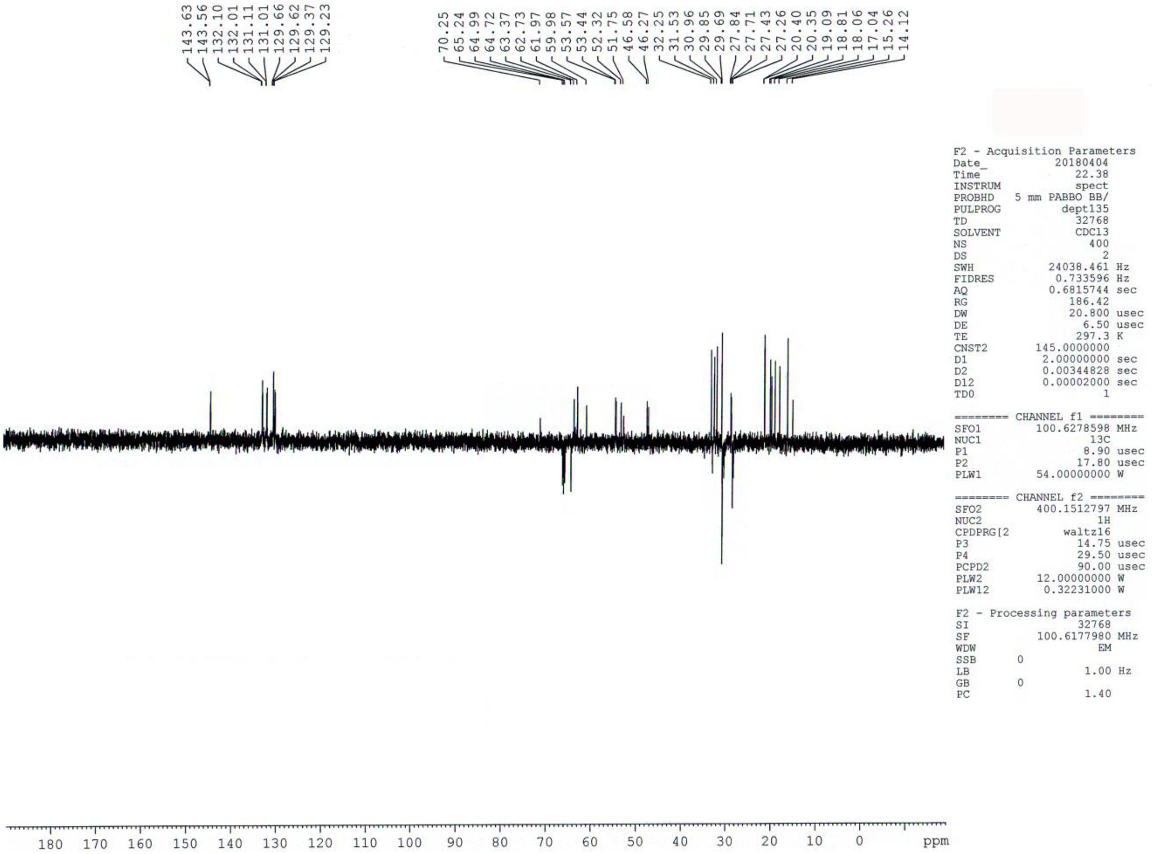

Figure S9: ^{13}C-DEPT-135 NMR of picolinamycin in $CDCl_3$

Figure S10

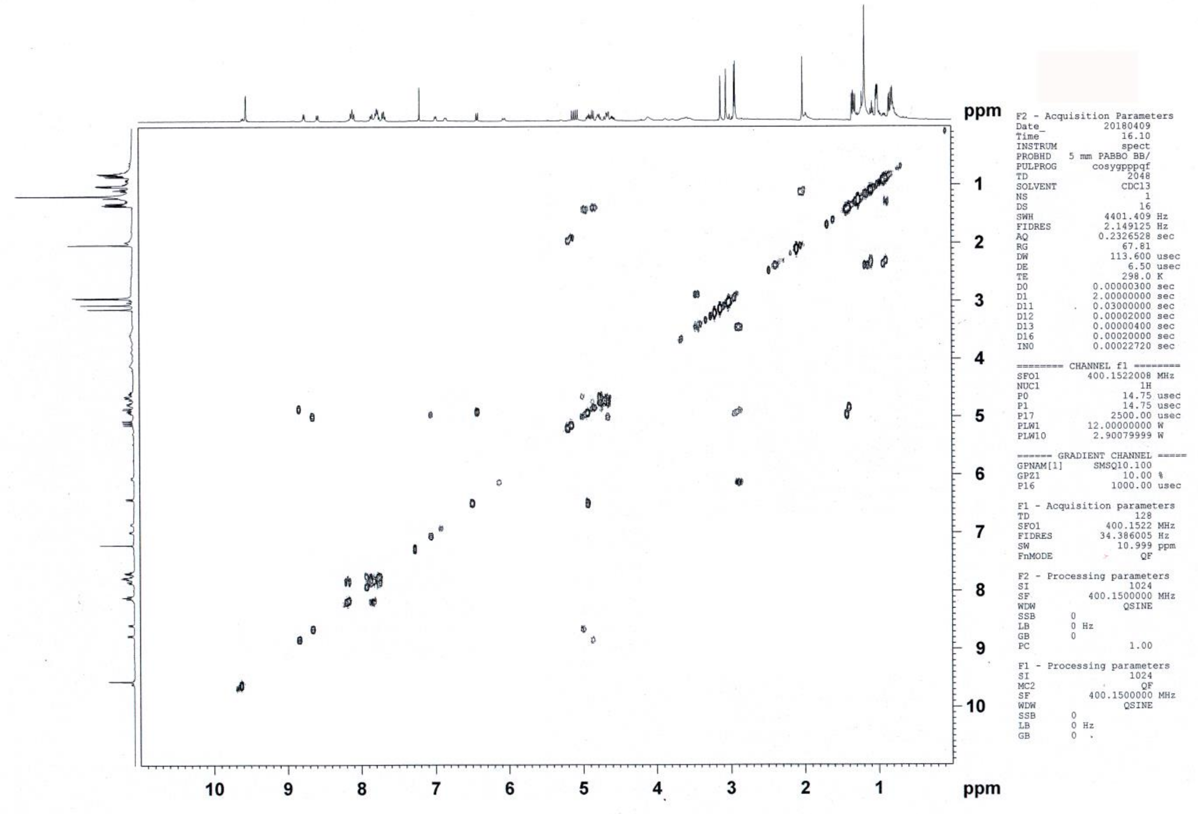

Figure S10: COSY NMR of picolinamycin in $CDCl_3$

Figure S11

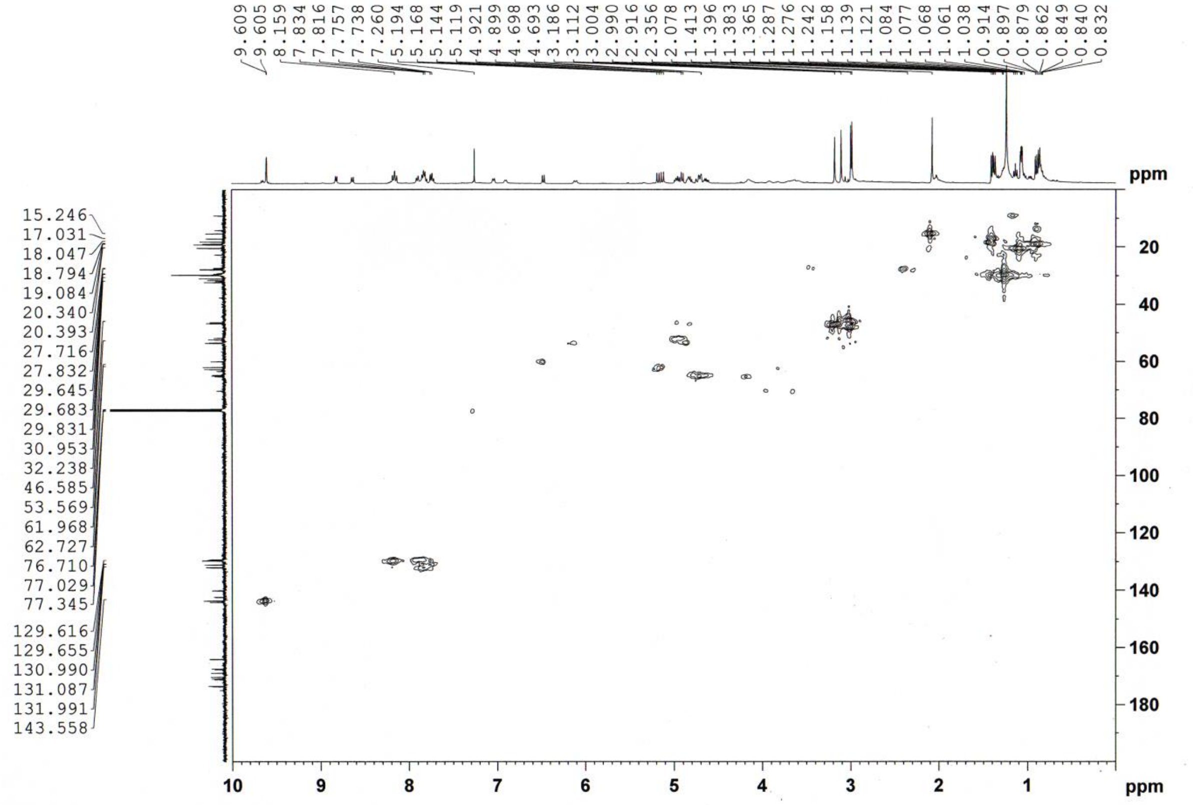

Figure S11: HMBC NMR of picolinamycin in $CDCl_3$
